# Variola, or Small-Pox

**Published:** 1838-07-04

**Authors:** N. Chapman

**Affiliations:** Professor of the Theory and Practice of Physic, in the University of Pennsylvania


### THE MEDICAL EXAMINER.

DEVOTED TO MEDICINE, SURGERY, AND THE COLLATERAL SCIENCES.

EDITED BY J. B. BIDDLE, M. D. AND M. CLYMER, M. D.

No. 14.] PHILADELPHIA, WEDNESDAY, JULY 4, 1838. [Vol. I.

### VARIOLA, OR SMALL-POX.

*By* N. Chapman, M. D., *Professor of the Theory and Practice of Physic, in the University of Pennsylvania.*

(Continued from page 204.)

In regard to the typhoid form of the disease, the treatment is different. This is a case of weak, inflammatory action, mixed up with a preponderance of congestion, and is to be managed on the principles, and by the remedies applicable to this condition under ordinary circumstances.

Evacuations of the alimentary canal are commenced with, though not to be carried to any great extent. An emetic, however, and purging in the beginning, should be practised, where there is heavy visceral turgescence, and the latter by calomel, especially. The loss of blood is, sometimes, of equal importance, and has been too much overlooked. Even when venesection is not allowable, topical bleeding may be safely and efficaciously employed. Early, however, we are sometimes compelled to resort to the means, calculated to sustain the vis vitæ, and which I need not again recapitulate minutely. To promote the filling and maturation of the pustules, a process languidly performed by the natural powers, or to restore the eruption, when it recedes, a combination of the sulphate of quinine and opium, or the carbonate of ammonia, or camphor, and wine whey, with the warm bath, constitute the best of our resources. Emetics have been proposed for the latter purpose, and might be serviceable. More certain, however, with either intention, are sinapisms and blisters, which are commonly put on the extremities, and, though indubitably serviceable, are less so than over the epigastrium. Let me here repeat, that, in all those affections seated in the stomach, much is attained in their feeble states, by arousing the energies of that viscus, and such applications are eminently appropriate to the occasion.

The case advancing with increased prostration of strength, an appeal is to be made to the freest use of the cordial and diffusible stimuli, at the head of which, in point of efficacy, is wine.

That the spirit of turpentine, as an internal remedy, should be useful at this period, is probable from analogy. Especially does it seem suited to those cases attended by petechiæ, vibices, and passive hæmorrhage. But I have no adequate experience with it. Much is said by Sydenham, under these circumstances, of the peculiar efficacy of the sulphuric acid.

As previously stated, it is an usual event, in both forms of this disease, at the full maturity of the eruption, for a fever to arise, or if it have continued, to increase considerably. Except puncturing the pustules, so as to remove the irritation of distention, I am not aware of any peculiarity in the established treatment. This is to be shaped to the nature of the case, and, inflammatory or typhoid, it is to be managed on similar principles, and by the same measures, as if such were the state originally. Extreme nervous irritation and inquietude supervene, in some instances. Combinations of opium and camphor are here very apposite, and so is Hoffman's anodyne liquor.

It was formerly stated, that, in the confluent disease, the state of the skin itself most materially influences the result. The condition is very similar to that of an extensive scald or burn, productive of nearly the same train of symptoms, and occasioning death probably in the same mode.

How far certain lotions, or other external applications, might in this case be beneficial, remains to be proved. We shall presently see, that camphor holds out some favorable promises. What should further encourage us to the employment of topical applications, is, their unequivocal efficacy in erisypelas. Many are the instances of this disease, kept up by irritation of the skin, that would terminate fatally, were it not thus allayed.

An application to the surface, of the mucilage of flaxseed, in the active inflammatory stage, I am sure would be proper, and perhaps in the subsequent or asthenic condition, the camphorated or Kentish ointment might be equally so—such being the most successful remedies, in the analogous affections to which I have alluded.

These are views, I believe, entirely original with myself, which I have had no opportunities of subjecting to the test of experience. The experiment, however, I cannot help thinking, is worthy of trial.

Of the minor parts of practice in small-pox, one which demands particular attention, is the prevention of those marks which are so detrimental to female beauty. As an exposure to the air is thought necessary to the production of the eruption, so our contrivances are directed to its exclusion.

Covering the face with fine cambric, spread with the mildest cerate, sometimes succeeds, and camphor is reputed to have the same effect. By Rosentein, a respectable writer, on the diseases of children, it is affirmed, that, if the skin be smeared over with camphorated ointment, the eruption will not take place on that part. It has recently been asserted, by some of the French writers,* that, puncturing the pustules with a lancet, and then touching them delicately with lunar caustic, they will be so destroyed, as to leave no marks. But this must be done on the first or second day of the eruption, it being nugatory afterwards. Neither of the expedients mentioned, have I tried, though they are certainly deserving of attention. My own practice has been to subdue inflammation as far as possible by cooling lotions, to open the pustules as soon as they fill, and wash them with milk and water. It appears, however, that the most effectual means is the exclusion of light. Experiments

* Velpeau and Meyreux.

made some few years ago, at New Orleans, if they can be relied on, and I know of no reason why they should be distrusted, are very satisfactory on this point. To try the effect of this expedient, a certain number of patients during the eruptive and maturative stages of the disease, were confined in a dark ward of the hospital, and not a pit or scar, or other deformity of the skin was left, though some of them had the disease most violently, even in the confluent form.*

Not the least serious of the local affections, is ophthalmia, causing very often blindness, by disorganizing the eyes. There is, however, as usually managed, nothing peculiar in the remedies. To prevent the inflammation, Rosentein advises, as very effectual, a bag of camphor to be kept before them. As to the ulcerated throat, an occasional incident also, the same detergent gargles, as in angina maligna, are to be directed. The best preventive, according to the writer just cited, is the free use of a camphorated gargle.

With this, I dismiss the subject of casual small-pox.

It has long been known, that by inoculation, this disease is disarmed of much of its violence, and of nearly all its danger. To such success had its management been reduced, under propitious circumstances, that it was computed, only one death, in five hundred cases, took place.

This is asserted by Woodville, as the result of his practice in the small-pox hospital of London, where he commanded every advantage. But, as we shall hereafter see, the average of mortality is considerably greater, though, still there is little to be dreaded from it when skilfully exercised. The late Professor Kuhn told me, that he had never lost a patient whom he inoculated—the same statement was made to me by Professor Physick, who added, that he learnt from Professor Rush, that he had lost only three, owing too, in part, to extraneous causes. Of the many whom I have had under my care, I do not recollect a solitary instance of death.

An explanation has already been given of the mitigation of the disease by this process. But it is alleged that it undergoes a further change from it. My allusion is to the deprivation it sustains of its infectious nature, or that in this state of amelioration, it ceases to communicate itself, through the medium of the atmosphere. Looking over the writers on the subject, I find, that while its capability under these circumstances is generally not doubted, there are many of the highest authority, particularly of the continent of Europe, by whom it is utterly denied, and others, who concede it in a slight degree only. These reports are entitled to the more weight, as proceeding from individuals whose attention was directed specially to the inquiry. On this point, I cannot come to any positive conclusion, from the want of adequate experience, it being, indeed, known to me only within a short period, as a matter of controversy. The least sustained view of it, however, is not improbable in itself, and is corroborated by some analogies. Examples are abundant of diseases indisputably contagious, and not at all infectious, that is, supplying a virus operative by contact or inoculation, without the generation or escape of any effluvia efficient to a similar end, among which, syphilis is conspicuous. But more pertinent illustration we have in the vaccine affection, which, while contagious, is totally devoid of infection, and, as it is probably modified small-pox, it may be rationally conceived, that, by inoculation, such a change is wrought in the latter disease, as to bring it into the same category. Granting the plausibility of all this, I still think that better evidence, than any hitherto adduced, is required to determine the question, and, from the deep interests it involves, in several relations, I trust it will command the most careful observations and experiments, by which alone, certainty can be obtained.

By whom the great discovery of inoculation was made, or among what people it originated, are questions of some obscurity. The Chinese, who reluctantly acknowledge a priority of claim to any thing, aver, that the practice of propagating small-pox, by the introduction of scales of the eruption into the nostrils, immemorially prevailed among them. Even if it did, it must be considered as giving the affection in the natural way, and not by inoculation. They denominated it sowing or disseminating the disease.

Tradition reports also, that the practice of artificially imparting small-pox, existed at a very remote period in Hindostan, and the execution of the office was committed to a particular tribe of Bramins, exclusively professing a knowledge of the art, who were delegated for the purpose, from certain religious colleges, to travel through the provinces. With a vast deal of ceremony and superstitious observance, the operation was performed by applying cotton soaked in the virus, on a small incision. This was really inoculation.

The credit of introducing this discovery into Europe, is generally accorded to the well known Lady Mary Wortley Montague, who had the experiment previously tried on her own son, in Constantinople, while she resided there, as the wife of the British Ambassador. The communication of this event, by her, was in 1717, and, five years afterwards, returning home, she had her daughter subjected to the same operation.

An attempt has been made, though unavailingly, to despoil her of this distinguished honour, by alleging, that the usage existed antecedently in Wales, and the Highlands of Scotland, as well as in several of the countries of the continent of Europe. It is said, that in Wales, it was called *buying the disease*, from a notion, that inoculation could not produce the proper effect, unless the person from whom the variolous matter was taken, received a piece of money, or some other article, in exchange for it, from those whom it was intended to infect.* Be it as it may, these averments against her claim, in my opinion, want the support of authentic evidence. It is however, true, that the practice of inoculation, or engrafting the disease, as it was then entitled, had been published in England, in 1714, by Timoni a physician of Constantinople, and by Pylanini of Venice, and the next year, by Kennedy, an English surgeon, who had visited Turkey. But

* Picton's Thesis.

*Woodside's Hist. of Inoculation, vol. 1, p. 42.

these publications attracted little or no attention, and cannot derogate from her pretensions.

As a means of preserving the exquisite beauty of their women, the practice prevailed with the Circassians, from whom the Turks borrowed it, and, seeing its efficacy constantly illustrated, the celebrated female to whom I have alluded, ventured on the trial just mentioned, and extended the benefits of the practice to her own country.

Like all innovations, or new discoveries, this encountered, in the commencement, a violent opposition as well from popular ignorance, as medical prejudices. It was arraigned as an impious attempt to counteract the visitations of Providence, not recollecting, according to such a notion, that every effort to arrest or mitigate the evils to which humanity is exposed, even the whole art of medicine itself becomes involved in the same criminalty. The pulpit thundered forth its anathemas against the sinfulness of the act, representing it, when voluntarily submitted to by an adult, in the enormity of suicide, and, in children, as an atrocious murder, for which the most awful responsibilities were hereafter incurred. There were not wanting some, who, in the phrensy of fanaticism, went so far as to pronounce it the invention of Satan himself, and its supporters as sorcerers and atheists arrayed against the will of God. By one of these infuriated preachers, it was, indeed, gravely declared, that inoculation is a very ancient art, first practised by the Devil on Job, and to which all the afflictions of that memorable sufferer were referred. This odd conceit, however, did not escape animadversion, and was very appropriately answered by a wag at the time, in the following doggerel rhymes:

"We are told by one of the black robe,
The Devil inoculated Job.
Suppose 'tis true, what he does tell,
Pray, neighbours, did not *Job* do well?"

Equally disreputable was the conduct of a part of our own profession, raising objections to it as futile as they were ridiculous, and absurd. But, happily, in this instance, there were enough of those master spirits, who are prompt to arise on such occasions, to put down ill-directed hostility, and to secure the ultimate triumph of a good cause!

Much praise is due to our own country for the establishment of the practice. In 1721, when it had only been adopted in England, in a few instances, and these chiefly on malefactors, whose punishment by death was thus commuted, and a load of prejudice still existed against it, Boylston, of Boston, a name deservedly high in the annals of American medicine, against the unanimous opinion of the other physicians of that city, and in direct contravention of an edict of the municipal authorities, carrying with it heavy penal consequences, had the intrepidity to inoculate 286 persons, of whom six only died. The disease was prevailing extensively at the time, and out of 5759 persons who took it in the natural way, 844 perished.

To escape from the conviction of the inestimable advantage of the process, after so successful an experiment, was impossible, and, when the result transpired in England, doubt and hesitation were rapidly removed* and the practice in no long time came to be generally adopted. Enlightened on the subject, perhaps, by this very intelligence, the reigning family at the period, stepped forward, and submitted to inoculation, to enforce it by the weight of their example, which no doubt, to a great extent, had an effect.

Treating of the events of 1723, Smollet, the historian, says, "That the practice of inoculation for the small-pox, was by this time introduced into England from Turkey. The Prince Frederick, the two Princesses, Amelia and Carolina, the Duke of Bedford and his sister, with many other persons of distinction, underwent the operation with success." By a comparison of dates, it will be seen, that this was two years after the report of Boylston. Yet, owing to circumstances which do not very clearly appear, the practice relapsed into discredit in England, to such a degree, indeed, that so lately as near the middle of the last century, it had fallen almost into neglect or disuse. By Meade, it is related, that, about this time, the most favourable reports of its success arrived from America and the West Indies, which, affording encouragement to its revival, a well regulated hospital for inoculation was opened in London—the college of physicians declared in its favour, and, henceforward, the practice became firmly established, till the introduction of vaccination, by which it has been nearly superseded.

(To be continued.)

* Ramsey's History, vol. 1, p. 272.

